# Association of Leptin With Obesity and Insulin Resistance

**DOI:** 10.7759/cureus.12178

**Published:** 2020-12-19

**Authors:** Ratan Kumar, Kheraj Mal, Muhammad Khalid Razaq, Mansoor Magsi, Muhammad Khizar Memon, Sidra Memon, Maham Noor Afroz, Humza F Siddiqui, Amber Rizwan

**Affiliations:** 1 Cardiology, Khairpur Medical College, Khairpur, PAK; 2 Cardiology, National Institute of Cardiovascular Diseases, Karachi, PAK; 3 Cardiology, Sheikh Zayed Medical College, Rahim Yar Khan, PAK; 4 Internal Medicine, Taluka Hospital Kandhkot, Kandhkot, PAK; 5 Internal Medicine, Liaquat University of Medical & Health Sciences, Jamshoro, PAK; 6 Internal Medicine, Jinnah Sindh Medical University, Karachi, PAK; 7 Medicine and Surgery, Jinnah Sindh Medical University, Karachi, PAK; 8 Oncology, Jinnah Post Graduate Medical Center, Karachi, PAK; 9 Family Medicine, Jinnah Post Graduate Medical Center, Karachi, PAK

**Keywords:** leptin, obesity, insulin resistance, diabetes

## Abstract

Introduction

Leptin, a hormone released by the body to regulate energy balance by inhibiting hunger, decreases fat storage in adipocytes. Leptin is thought to play some role in obesity and insulin resistance. In this study, our aim is to see the association of leptin with obesity and insulin resistance.

Methods

This case-control study was conducted in a tertiary care hospital in Pakistan from January 2020 to April 2020. Ninety-two participants with BMI greater than 25 kg/m^2^, with no known comorbidities were enrolled in the study after informed consent. Ninety-two participants, who came to the outpatient department without a history of chronic disease, with BMI less than 25 kg/m^2^ were enrolled as a control group. Data were collected via self-structured questionnaires. Their blood was drawn and sent to the laboratory for cholesterol levels, insulin resistance and leptin levels.

Results

Serum leptin levels (51.24 ± 18.12 vs. 9.10 ± 2.99: p-value, < 0.0001), serum cholesterol levels (198.2 ± 32.1 vs. 151.2 ± 21.2, p-value < 0.0001) and insulin resistance (7.9 ± 2.1 vs. 6.3 ± 1.9, p-value < 0.0001) were higher in obese patients.

Conclusion

As per the results of this study, obesity was associated with increase serum leptin levels and insulin resistance. Further multi-centric studies are required to prove the possible relationship, which might help devise plans to manage obesity.

## Introduction

Leptin is a hormone released by adipose tissue and small intestine cells to regulate energy balance in proportion to triglycerides via certain neural pathways mainly hypothalamus to inhibit hunger which in turn decreases fat storage in adipocytes [1-4]. Because of its mode of action, leptin is thought to play some role in the development of obesity and insulin resistance [2,3,5].

Obesity is a complex phenomenon in which there is deposition of excess amount of fat. It increases the risk of cardiovascular diseases (CVDs), stroke, osteoarthritis, diabetes, and reproductive functions [6]. Globally, the prevalence of obesity has steadily increased during the past 30 years [7]. Izquierdo AG et al., in his review article, suggested that obese people have elevated serum levels of leptin as explained by leptin resistance in such individuals [8]. This resistance leads to failure of hunger suppression and increased food intake, which ultimately leads to obesity [9]. Obese individuals are also more likely to develop insulin resistance and have high cholesterol levels, which can lead to development of numerous chronic illnesses [5]. Obesity, leptin resistance and insulin resistance are interrelated. Studies have suggested that hyperinsulinemia can be a potential cause of leptin resistance and hence obesity, eventually leading to metabolic syndrome in such individuals [10].

Although global research has been done on the association between leptin, obesity and insulin resistance, however, as per our knowledge, there is no local data available regarding this subject in the Pakistani population.

## Materials and methods

This case-control study was conducted in a tertiary care hospital in Pakistan. Data were collected from the attendant of patients visiting the outpatient department of the medicine ward from January 2020 to April 2020. Ninety-two participants with BMI greater than 25 kg/m^2^, without any known comorbidities such as hypertension, diabetes or previous history of cardiovascular events were enrolled in the study after informed consent. Ninety-two participants, who came to the outpatient department without a history of chronic disease, with BMI less than 25 kg/m^2^ were enrolled as a control group.

After the inclusion of participants in the study, their demographics such as age, BMI, gender, and smoking history were noted in self-structured questionnaires. Their blood was drawn and sent to the laboratory for cholesterol levels, insulin resistance and leptin levels. The serum levels of leptin were measured using a radioimmunoassay (RIA) (normal range: 2.5-21.8 ng/mL) [11].

Statistical analysis was done using SPSS, v. 23.0 (IBM Corporation, Armonk, NY). Continuous variables including age, insulin resistance, body mass index, cholesterol levels and leptin levels were analyzed via descriptive statistics and were presented as means and SDs while categorical variables such as smoking history were presented as percentages and frequencies. T-test and Chi-square were applied as appropriate. p-value of less than 0.05 meant that the difference between the groups is significant and the null hypothesis is void.

## Results

Leptin was significantly higher in patients with obesity (51.24 ± 18.12 vs. 9.10 ± 2.99: p-value < 0.0001). Cholesterol (198.2 ± 32.1 vs. 151.2 ± 21.2, p-value < 0.0001) and insulin resistance (7.9 ± 2.1 vs. 6.3 ± 1.9, p-value < 0.0001) were also significantly higher in patients with obesity (Table 1).

**Table 1:** Comparison between obese and non-obese patients.

Characteristics (mean ± SD)	BMI more than 25 kg/m^2^	BMI less than 25 kg/m^2^	p-value
Age (years)	49 ± 12	47 ± 11	Not significant
Gender			
Male	35 (38.0%)	47 (51.0%)	Not Significant
Female	57 (62.0%)	45 (49.0%)	
Body Mass Index (kg/m^2^)	28.2 ± 3.1	21.1 ± 1.5	< 0.0001
Insulin Resistance (mU/L)	7.9 ± 2.1	6.3 ± 1.9	< 0.0001
Cholesterol Levels (mg/L)	198.2 ± 32.1	151.2 ± 21.2	< 0.0001
Smoking Status (%)	32 (34.7%)	26 (28.2%)	
Total Leptin (ng/mL)	51.24 ± 18.12	9.10 ± 2.99	< 0.0001

Leptin levels were above normal level in 52.2% obese patients compared to 13.1% in non-obese patients. The odds ratio was 7.7 (3.49-15.11, 95% CI) (Table 2).

**Table 2:** Leptin levels in obese vs. non-obese patients. CI, confidence interval; OR, odds ratio.

Leptin (ng/mL)	Obese Patients (n = 92)	Non-Obese patients (n = 92)	95% CI	OR
Normal Level (below 21.8 ng/mL)	44 (47.8%)	80 (86.9%)	3.49-15.11	7.27
Increased Level (above 21.8 ng/mL)	48 (52.2%)	12 (13.1%)		

## Discussion

Obesity and insulin resistance are the primary components of metabolic syndrome and these are the main modifiable risk factors for cardiovascular disease. Positive correlation between obesity and insulin resistance has been well documented. However, they also share another common link in the form of hyperleptinemia. Studies have observed that obesity causes high levels of leptin, which acts as a pro-inflammatory cytokine and amplifies the process of insulin resistance [12].

This study demonstrated significantly higher levels of leptin in obese patients (51.24 ± 18.12) than non-obese patients (9.10 ± 2.99) with p-value < 0.0001. Moreover, greater than 50% of the obese patients had leptin levels above 21.8 ng/ml while only 13.1% non-obese showed these levels. Minocci et al. also reported similar findings that increased BMI was associated with increased leptin levels. These levels were directly proportional to subcutaneous fat and were inversely proportional to abdominal fat index and/or waist-hip ratio [13]. This is the plausible explanation that might explain why leptin levels were higher in females rather than in males. As females acquire fat deposition in subcutaneous depot and males acquire more visceral fat [14]. This sexual dimorphism was also found in another study conducted by Couillard and his colleagues. They demonstrated that high levels of leptin are associated with adipose cell hypertrophy, which is seen more in females as compared to adipose tissue hyperplasia seen commonly in males [15].

As leptin reduces appetite and body weight, the paradoxical coexistence of obesity and hyperleptinemia suggests the pathology of “leptin resistance” [16]. Leptin resistance can be due to defect in intracellular mechanism or due to impairment in transport through blood-brain barrier. Several pathways related to the development of leptin resistance have been studied in animal models such as the fat mass and obesity-related gene, oestradiol (E2) and peroxisome proliferator-activated receptor γ, phosphodiesterase-3B (PDE3B)-cAMP- and Akt-pathways of leptin signalling in the hypothalamus [17,18]. These findings open the door to understanding the pathophysiology of leptin resistance in humans. On the other hand, congenital leptin deficiency is associated with hyperphagia and early-onset obesity. This goes along with the basic physiology of leptin and its effects on hypothalamus as an anti-obesity hormone [19,20].

Insulin resistance (IR) is defined as the inability of the known quantity of insulin hormone to impart its effects on the body tissue as it does in the normal population [21]. In this study, we found out IR was 7.9 ± 2.1 in obese patients which is much higher than 6.3 ± 1.9 in non-obese patients. The pathophysiology behind obesity-induced IR and diabetes mellitus type 2 (DM2) is thought to be due to systemic inflammation. Proinflammatory cytokines such as Interleukin 1 beta (IL-1β) have been implicated to induce IR through the activation NLR family pyrin domain containing 3 (NLRP3) inflammasome [22].

Leptin also has a role in inflammation. High levels of leptin are positively associated with systemic markers of inflammation such as serum amyloid A (SAA), C-reactive protein (CRP) and osteopontin as well with markers of systemic oxidation such as thiobarbituric acid reactive substances (TBARS) [23]. This observation was further proved by another study where Uslu et al enrolled 85 DM2 patients and found an increased level of leptin (a proinflammatory adipocytokine) and decreased level of adiponectin (an anti-inflammatory cytokine). The author also suggested the application of leptin and adiponectin ratio as a CVD risk factor in DM2 patients [24].

To the best of knowledge, this is the first local study that studies the correlation between body mass index and leptin level. There are certain limitations for the study as well. First, since it is a case-control study, no definite association can be established. Also, since the study was conducted in single-center, the sample diversity was reduced.

Leptin is an important marker and may play a role in obesity, cardiovascular disease, inflammation, insulin resistance and diabetes mellitus type 2. It is important that leptin levels are periodically monitored in high-risk patients and appropriate management to reduce leptin levels is done.

## Conclusions

In this study, high levels of leptin were associated with increased BMI and insulin resistance. Leptin is the hallmark of obesity and is a major appetite suppressant, although no effective obesity therapy based on this hormone has been developed till date. Role of leptin in obesity and insulin resistance and its effectiveness in the management of obesity is under many studies. Physicians should be encouraged to include leptin levels in the management of obesity and other metabolic syndromes.
